# Promoting physical distancing during COVID-19: a systematic approach to compare behavioral interventions

**DOI:** 10.1038/s41598-021-98964-z

**Published:** 2021-09-30

**Authors:** Tessa F. Blanken, Charlotte C. Tanis, Floor H. Nauta, Fabian Dablander, Bonne J. H. Zijlstra, Rick R. M. Bouten, Quinten H. Oostvogel, Meier J. Boersma, Maya V. van der Steenhoven, Frenk van Harreveld, Sanne de Wit, Denny Borsboom

**Affiliations:** 1 Department of Psychology, University of Amsterdam, 1018 WT Amsterdam, The Netherlands; 2 Research Institute of Child Development and Education, University of Amsterdam, 1012 WX Amsterdam, The Netherlands; 3 Focus Technologies B.V., 5657 EW Eindhoven, The Netherlands; 4 Smart Distance Lab, 2353 NM Leiderdorp, The Netherlands; 5 National Institute for Public Health and the Environment (RIVM), 3721 MA Bilthoven, The Netherlands

**Keywords:** Health policy, Public health

## Abstract

In the wake of the COVID-19 pandemic, physical distancing behavior turned out to be key to mitigating the virus spread. Therefore, it is crucial that we understand how we can successfully alter our behavior and promote physical distancing. We present a framework to systematically assess the effectiveness of behavioral interventions to stimulate physical distancing. In addition, we demonstrate the feasibility of this framework in a large-scale natural experiment (*N* = 639) conducted during an art fair. In an experimental design, we varied interventions to evaluate the effect of face masks, walking directions, and immediate feedback on visitors’ contacts. We represent visitors as nodes, and their contacts as links in a contact network. Subsequently, we used network modelling to test for differences in these contact networks. We find no evidence that face masks influence physical distancing, while unidirectional walking directions and buzzer feedback do positively impact physical distancing. This study offers a feasible way to optimize physical distancing interventions through scientific research. As such, the presented framework provides society with the means to directly evaluate interventions, so that policy can be based on evidence rather than conjecture.

## Introduction

COVID-19 holds the world in its grip. December 31st 2020, a year after the first statement about a new virus reached the WHO, over 80 million confirmed COVID-19 cases and 1.78 million deaths in 222 countries have been reported^1^. The virus continues to spread, and many countries suffer from a second wave of infections. As long as the world was awaiting the development, production, and distribution of effective vaccines, we only had one weapon at our disposal in our battle against the Coronavirus: our behavior^2^.

The World Health Organization (WHO) increasingly and repeatedly stresses the importance of engaging in ‘safety behaviors’, which include washing hands, wearing face masks, and physical distancing. Ultimately, physical distancing is among the most important of the recommended safety behaviors^3^ because, as complex as the virus may be, transmission of the virus requires an infectious person to be in physical proximity to other people. Therefore, in absence of a vaccine, it is pivotal to reduce the number of contacts between people that are in such physical proximity^4^.

Despite its universally acclaimed importance, it is unclear how we can optimally stimulate physical distancing through behavioral interventions. Whereas the effectiveness of policy measures to reduce mobility has been investigated (e.g.,^5^), the effects of more subtle behavioral interventions largely remain a matter of speculation and debate. One of the most striking examples of this debate concerns the behavioral effects of wearing face masks. Although early on in the pandemic extensive experimental research was conducted on the protective function of face masks (e.g., see^6^ for a review), studies investigating the behavioral effect of wearing face masks on physical distancing remained scarce.

In absence of such experiments, a variety of positions on the behavioral effects of face masks was defended. Policy makers and the World Health Organization expressed their concern that face masks could give people a false sense of security^7,8^, potentially leading to reduced adherence to other preventive regulations such as physical distancing. The phenomenon that people adjust their behavior in response to the perceived risk is more generally known as the Peltzman effect^9^, which could explain such compensation behavior. In contrast, others argued that face masks remind people to implement safety behaviors, thereby increasing compliance with COVID-19 regulations (e.g.^10^). Notably, even after evidence on the medical effects of face masks had accumulated, the debate around the implementation persisted due to the uncertainty of the behavioral effects of face mask policies (e.g.^11^).

Over the last year some studies have investigated the effect of face masks on physical distancing. Using an experimental set-up it was shown that generally people keep more distance from a confederate when the confederate was wearing a face mask compared to wearing no mask^12–14^, although some evidence was also found for the reverse pattern^15^. In observational studies it was moreover shown that mandatory wearing of face masks is positively associated to physical distancing^10^. However, through observational studies it is difficult to disentangle these findings from other confounding factors, such as incidence rates and public health messaging. The current paper adds to the literature on the relation between wearing face masks and physical distancing behavior, specifically by experimentally investigating the effect of wearing a face mask, as opposed to the effect of seeing someone else wear a face mask, on physical distancing.

The debate on mandatory face masks exemplifies how existing scientific work in psychology and behavioral science can provide support for opposing accounts, confusing and occasionally paralyzing policy. At the same time COVID-19 challenges our behavior in ways that have not been anticipated or assessed in previous research, thereby questioning the relevance of existing and behavioral science literature to this novel crisis^2,16^. This has spurred several investigations of how psychological factors can facilitate a better adherence to safety behaviors, such as the role of emotions^17,18^, reasoning^19^, and the effect of public health messaging^20–23^. However, it is important to note that most of these studies focus on self-reports and intentions rather than on actual behavior. Additional experiments are especially important as the external validity and generalizability of any one particular experiment is limited, requiring to build up an evidence base consisting of different experiments. The novelty of the COVID-19 pandemic thus warrant only one way forward: to assess the effect of behavioral interventions on physical distancing behavior experimentally.

**Figure 1:**
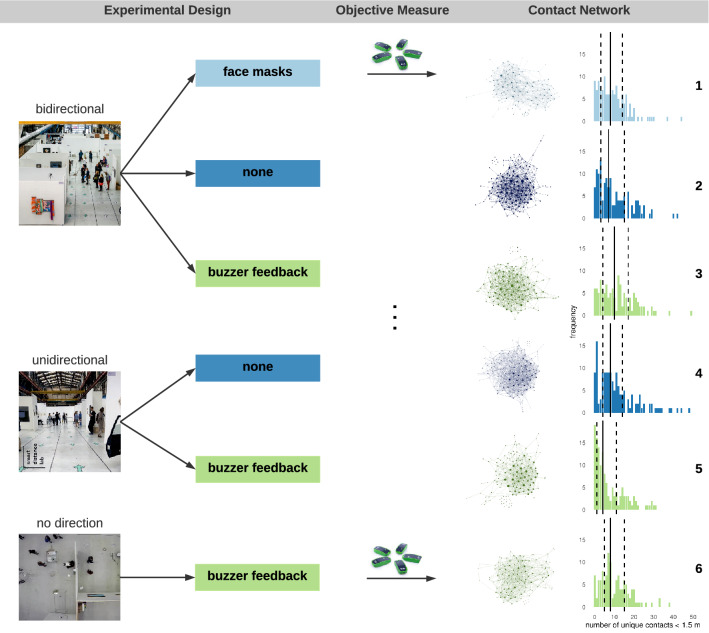
Representation and application of the novel methodological framework. The framework consists of (1) an experimental design; (2) objective physical distance measures; and (3) a contact network representation. Here, we show the framework applied at the art fair, where we (1) varied walking directions and supplementary interventions; (2) measured physical distance using a Social Distancing Sensor with ultra-wideband technology; and (3) constructed networks for each condition.

Investigation of the effect of behavioral interventions on physical distancing is hampered, however, by the lack of suitable methodologies that connect behavior to relevant epidemiological properties. In the current paper we describe, implement, and evaluate a novel methodological framework suited to directly test the effectiveness of different behavioral interventions on physical distancing. The framework is shown in Fig. 1 and involves (1) an experimental design; (2) objective measures of physical distancing; and (3) the representation of people (nodes) and their contacts (links) in a contact network. The proposed methodological framework enables us to evaluate the effectiveness of behavioral interventions on physical distancing through network representations^24^.

We implemented this novel framework in a first experimental study during a 3-day art fair in Amsterdam. As shown in Fig. 1, in the *experimental design* we implemented walking direction interventions (bidirectional; unidirectional; no direction) and supplementary interventions (face masks; buzzer feedback when within 1.5 m of another visitor; no supplementary intervention) in 2-h time slots. Note that at the time of the art fair in August 2020, face masks were not mandatory in The Netherlands, allowing us to incorporate face masks as an experimental condition. See “Methods” section for details. We *objectively measured* physical proximity between visitors using a Social Distancing Sensor (SDS) which utilizes ultra-wideband technology. Whenever two visitors came within 1.5 m from each other, the contact was logged. Subsequently, we used the logged contacts to construct the *contact networks* of visitors for each experimental condition.

In constructing the networks, we collapsed the contacts over time without losing important information, because only primary infections can be transmitted within a 2-h time frame, and therefore only direct contacts are virologically relevant. In other words, if a visitor is infected during the event, this visitor cannot subsequently infect others due to the incubation period, and as a result the order in which people make contacts can be disregarded. In accordance, here we only focus on the direct paths between nodes in the network, which specify the number of unique contacts between visitors and are encoded in the *degree distribution*. Finally, we use innovative statistical network methodology, based on an adaptation of Van Duijn^25^ to undirected networks, which allows us to statistically test differences in the degree distribution (i.e., the distribution of the number of contacts) across experimental conditions.

In short, the proposed methodology enables us to assess the effects of interventions directly at the level of behavior. The methodology is straightforward to implement and provides society with the means to directly evaluate interventions, so that policy can be based on evidence rather than conjecture.

## Results

### Descriptives

#### Visitors

Over the course of 3 days 787 people visited the art fair, of whom 639 (81.2%) participated in our study and wore a Social Distancing Sensor, see Table 1. We note that on Saturday 29th and Sunday 30th the art fair opened at 11:00 before the experiment started at 13:30. 74 and 132 visitors, respectively, had entered the art fair between 11:00 and 13:30, of whom some had already left before the experimental condition started. Therefore, some of the 787 visitors did not participate in our studies, and thus the actual percentage of visitors that wore the SDS is higher than the reported 81%. For 392 (61.3%) visitors demographic information was available as they completed a questionnaire upon purchasing their ticket. These visitors were 42 ± 16 (mean ± SD) years old (14–89 years), 225 (57.3%) were female, and 335 (85.5%) of them completed a higher education.

**Table 1:** Descriptives per condition.

Condition			n	Duration (min)	Pre Q (%)	Post Q (%)	Age mean (± SD)	Males: females n (%): n (%)	Number of contacts < 1.5 m			
									Range	Mean (± SD)	Median	IQR
1	08/28 13:30–15:30	Face mask bidirectional	130	120	66 (51%)	41 (32%)	41.3 (± 17.0)	32 (48%) 34 (52%)	0–44	9.3 (± 7.8)	8	3–14
2	08/28 15:30–17:30	No intervention bidirectional	137	120	77 (56%)	35 (32%)	43.2 (± 15.1)	34 (44%) 43 (56%)	0–42	9.8 (± 8.5)	7	3–15
3	08/28 17:30–19:30	Buzzer bidirectional	122	120	66 (54%)	24 (20%)	43.9 (± 13.6)	24 (36%) 42 (64%)	0–49	11.7 (± 8.9)	10	4–17
4	08/29 13:30–15:30	No intervention unidirectional	147	120	86 (59%)	40 (27%)	43.4 (± 16.0)	38 (45%) 46 (55%)	0–48	11.0 (± 9.9)	8	4–14
5	08/29 16:00–18:00	Buzzer unidirectional	137	120	78 (57%)	32 (23%)	43.4 (± 16.9)	31 (40%) 46 (60%)	0–31	7.3 (± 7.8)	4	1–11
6	08/30 13:30–15:30	Buzzer no direction	123	120	62 (50%)	18 (15%)	42.2 (± 15.7)	23 (37%) 39 (63%)	0–38	10.3 (± 7.5)	8	5–15
7^a^	08/30 15:30–16:30	Buzzer no direction	146	60	79 (54%)	22 (15%)	42.8 (± 16.1)	31 (39%) 48 (61%)	0–28	8.2 (± 6.1)	7	4–11
8^a^	08/30 16:30–17:30	No intervention no direction	102	60	51 (50%)	3 (3%)	39.8 (± 16.0)	20 (39%) 31 (61%)	0–30	5.9 (± 5.3)	5	2–8

Information on age and sex is based only on visitors who completed the pre-questionnaire and provided this information (n = 3 did not wish to disclose their sex). Number of visitors in each condition don’t add to N = 639, as some people stayed for more than one condition.

^a^Condition lasted only 60 min and was excluded from analyses.

### Interventions

From the conditions shown in Table 1, we only selected the six conditions that lasted 2 h (visualized in Fig. 1, see “Methods” for a more elaborate explanation). To evaluate the effectiveness of each behavioral intervention in isolation (*face masks*, *walking directions*, *buzzer*) we select two conditions that differ only in whether the behavioral intervention of interest is implemented, allowing us to estimate the effect of that intervention.

#### Face masks

To examine the effect of wearing face masks on physical distancing, we compared the contact networks for the face mask condition with the no supplementary intervention condition on the first day (condition 1 vs. 2 in Fig. 1). As can be seen from Table 1, visitors had a median of 8 unique contacts (IQR = 3–14, range = 0–44) in the face mask condition, whereas visitors in the no supplementary intervention condition had a median of 7 unique contacts (IQR = 3–15, range = 0–42). Detailed analysis of the probability of visitors forming contacts in these two conditions—taking the network structure of the data into account (see “Methods”)—did not provide evidence for an effect of wearing face masks on distancing (*OR* = 1.05, 95% Credible Interval [0.81, 1.33]). These results indicate that, if there are differences in the number of contacts when wearing face masks compared to when not wearing them, these differences are likely to be small. In other words, face masks do not appear to facilitate or inhibit physical distancing.

#### Walking direction conditions

We performed two comparisons. First, we evaluated the effect of unidirectional walking directions versus no walking directions by comparing the buzzer conditions on day two and three (condition 5 vs. 6). On day 2 with unidirectional walking directions, visitors had a median of 4 unique contacts (IQR = 1–11; range = 0–31), whereas on day 3 without walking directions, visitors had a median of 8 unique contacts (IQR = 5–15; range = 0–38). The probability of visitors forming contacts was substantially higher in the condition with no walking directions compared to the condition with unidirectional walking directions (*OR* = 1.66, 95% CI [1.25, 2.17]).

Second, we evaluated the effect of bidirectional versus unidirectional walking directions, by comparing the no supplementary intervention conditions on days 1 and 2 respectively (condition 2 vs. 4). During bidirectional walking directions, visitors had a median of 7 unique contacts (IQR = 3–15; range = 0–42), whereas during unidirectional walking directions, visitors had a median of 8 unique contacts (IQR = 4–14; range = 0–48). The probability of visitors forming contacts was about the same in these two conditions (*OR* = 0.99, 95% CI [0.75, 1.26]). This indicates that, if there are differences in the number of unique contacts visitors had when given bidirectional versus unidirectional walking directions, these are likely to be small. Thus, the implementation of walking directions appears to improve physical distancing, but the differential effects of unidirectional and bidirectional implementations were not statistically significant.

#### Buzzer

We investigated whether notifying visitors of violations of the 1.5 m rule through a buzzer reduces the number of contacts by comparing the buzzer condition to the no supplementary intervention condition (condition 2 vs. 3). The median number of unique contacts was 10 (IQR = 3–15; range = 0–42) for visitors in the buzzer condition and 7 (IQR = 4–17; range = 0–49) for visitors in the no supplementary intervention condition. The probability of visitors forming contacts was slightly higher in the buzzer condition than in the no supplementary intervention condition (*OR* = 1.24, 95% CI [0.95, 1.55]). The credible interval around this estimate suggests that buzzers likely do not promote physical distancing, and may actually make it worse. In interpreting this effect, it is however important to note that on this day visitors did not receive an instruction on how the buzzer worked, and feedback occurred only 2 s after the contact was made.

After improvements were made to the buzzer intervention (i.e., by implementing a short demonstration and removing the delay between registration of distance violation and feedback) the buzzer did promote distancing (conditions 5 vs. 4). With adequate instructions and immediate feedback, the median number of unique contacts of visitors in the buzzer condition was 4 (IQR = 1–11; range = 0–31) compared with a median of 8 (IQR = 4–14; range = 0–48) unique contacts in the no supplementary condition. The probability of visitors forming contacts was markedly higher in the no supplementary intervention condition, as compared to the buzzer conditions (*OR* = 1.43, 95% CI [1.06, 1.91]). This suggests that buzzer feedback can promote physical distancing, but that its effect is dependent on the instructions and immediate feedback.

## Discussion

In this paper we introduced and demonstrated a practical methodology to assess the effect of behavioral interventions on physical distancing. The methodology combines systematic variation of interventions in an experimental design with contact network assessment through wearable sensors. Analyzing experimentally induced changes in the contact network allows researchers to systematically track the effect of behavioral interventions and, as such, brings epidemiologically relevant human behavior within reach of the scientific method. Because it uses assessment of intervention effects at the level of behaviors that are known to affect virus transmission, the introduced methodology can be implemented to bridge the gap between behavioral science and epidemiology. Therefore, this type of research can directly inform policy concerning the mitigation of virus spread.

The implementation of the proposed methodology in the current experimental design may have several policy implications. First, consistent with recent findings from observational research^26^, we observed no significant effects of wearing face masks on physical distancing. This suggests that risk compensation, also known as the Peltzman effect^9^, is not operative in the case of face masks. Interestingly, however, previous studies showed that seeing someone else wear a face mask does influence physical distancing behavior^12–15^. Together, these findings also underscore the need to execute dedicated empirical research in different contexts to evaluate behavioral hypotheses, as it illustrates how *prima facie* plausible claims about human behavior may miss the mark^16^. At the same time, it is important to note that while the Peltzman effect may not play a role in mandatory wearing of face masks, it could very well have a profound effect on the course of the pandemic, e.g., through its effects on risk taking after vaccination^27^.

Second, our analysis clearly shows that implementing walking directions lowers the probability of contacts between visitors in the relevant conditions. We hypothesize that this benefit is counteracted by the fact that the movement patterns of other visitors are relatively unpredictable in a free movement scheme, so that distancing requires more effort and attention, and accidental collisions occur more frequently. The latter hypothesis could be investigated by subjecting movement patterns isolated from camera data^28^ to quantitative pedestrian modelling^29^. In comparing different walking directions, we did not observe differential effects of uni- versus bidirectional schemes. Given the dynamics of viral transmission, unidirectional walking directions should be preferred because they mitigate the probability of people facing each other directly; however, in situations where a unidirectional scheme is not available, a bidirectional scheme appears to be a good alternative. The implementation of walking directions is feasible in almost all public spaces and the current study directly supports this policy.

Third, our study opens the possibility of utilizing schemes that use sensor data information to provide people with feedback on their behavior. The current study used a style of feedback most closely related to an alarm: a pattern of light and a buzzer emitted when potential danger is encountered. While this style of feedback does seem to have a positive effect on adherence to distancing guidelines in the current study, its implementation is potentially challenging. Specifically, effective use of sensors seems to depend on adequate instruction and immediate feedback. In the absence of this guidance, on the first day of our study, visitors tended to try out the sensors and as such inadvertently came within 1.5 m of another visitor. This may have obscured the positive effects of feedback on subsequent distancing. After the first day, we countered this effect through a quite elaborate instruction where visitors were invited to try out the sensor on a trial sensor that was not included in the data analysis. However, in many cases such an instruction would be impractical, as it would take time and may lead to queue formation. One last consideration is that wearing a sensor, even without active feedback, could make people aware that they are being measured, thereby influencing their behavior, also known as the *mere-measurement* effect^30^.

Interestingly, in the current study the alarm scheme setup warns, but does not reward individuals for keeping distance. Other more rewarding ways to organize feedback may be more engaging (e.g., using gamification by letting people win various awards if they keep their distance) and informative for the user (e.g., many people asked for feedback on how many contacts they made). Clearly, there is a great potential for learning theory (e.g., reinforcement learning) to inform this process^31^; next to encouraging distancing upon receiving feedback, a feedback system could promote learning to distance effectively and automatically/efficiently.

The present study has yielded experimental support for policy advise concerning physical distancing. However, some limitations warrant attention. First, as we conducted the experiment in a naturalistic setting, we were not in a position to randomize the visitors to time slots. Since participants were not aware of the interventions that were implemented in each time slot, and as the exhibition in the art-fair was the same for all days, a priori differences between visitors are not expected; yet, in future research it is important to extend the design to experimental setups that utilize random assignment to bolster causal conclusions. Second, as we were unable to counterbalance all conditions, the design may yield some confounds; for example, based on the time of day a condition was measured. Third, as visitors could stay as long as they liked, some visitors were part of multiple conditions creating some dependency in the data. Fourth, as the experiment was conducted at an art fair, the visitors are not a random sample from the population and the results may not generalize to the general population or other settings. Similarly, the effectiveness of certain measures implemented at an art fair, may not readily translate to other contexts, such as for example a supermarket. At the same time, a naturalistic design has the advantage of high ecological validity. Ultimately, as different contexts draw different crowds and behavior is affected by one’s environment, we should replicate the interventions across various contexts (e.g., in a supermarket, or at a football stadium) to determine which interventions work best in which contexts.

In conclusion, the presented research methodology has a broad range of applications and can be used to assess behavioral interventions quickly and effectively. The current study provides a template for how such research can be organized. Our analysis code and data (reported separately in Tanis et al.^28^) are available for use in future studies (https://osf.io/u9avy/). We hope that this framework will stimulate the behavioral science community to engage with the problem of mitigating virus spread through behavioral interventions.

## Methods

### Participants

The participants in our naturalistic study were visitors of Smart Distance Lab: The Art Fair (www.smartdistancelab.nl). Upon purchasing their ticket, participants were informed that a study would take place during the art fair and that different interventions would be implemented. It was not told, however, which interventions would be implemented when. Participants could select a 30 min time slot themselves (see procedure), which allowed us to ensure that the visitors were sufficiently distributed across experimental conditions. As participants could select their own time slot, the design is a natural experiment rather than a randomized controlled trial.

### Design

The experiment took place at an art fair in De Kromhouthal in Amsterdam, see Tanis et al.^28^ for specifics on the layout. Across experimental conditions, we varied two sets of interventions: walking directions and supplementary interventions. Walking directions varied across the 3 days: bidirectional, unidirectional, and no walking directions respectively, see also Fig. 1. Within days, we varied supplementary interventions: face masks, a buzzer that indicated if one came within 1.5 m of another visitor, no supplementary intervention. It is important to note that when the art fair took place in August 2020, wearing face masks was not obligatory in The Netherlands, and only very few people wore face masks at the time. Of course, if visitors wanted to wear a face mask they were allowed to at all times. While we planned to cross all levels, we deviated from this plan, see “Practical and technological problems”.

### Materials

#### Questionnaires

We administered a pre- and post-questionnaire. The pre-questionnaire included items on demographics, potential coronavirus infection, worries about consequences of the virus, physical distancing and face masks. The post-questionnaire focused on physical distancing behavior during the art fair, and the experience of the event. In this paper, we only use visitors’ demographics such as age and gender, see Tanis et al.^28^ for an elaborate description of both questionnaires.

#### Social distancing sensor

In response to the COVID-19 pandemic Focus Technologies B.V. (www.findfocus.nl) together with Sentech B.V. (www.sentech.nl) have developed the Social Distancing Sensor. This sensor is able to determine the distance between itself and other Social Distancing Sensors using ultra-wideband technology with an accuracy of up to ten centimeters. Every sensor has a unique ID and registers how often a violation has occurred with every other sensor. An access point and a computer application are used to manage the system and set settings. Every time a sensor is within reach of the access point, it communicates all distance violations to the access point after which it is written to a central database. A distance violation between two sensors is logged on both sensors and is therefore written to the database twice. The sensor is able to provide direct feedback to the user using a flashing light, a buzzing sensation or a beeping sound. In this research only the light and buzzer are used. To accommodate visitors from the same household, multiple sensors can be paired after which violations of the 1.5 m distance between these specific sensors are not logged to the database and no feedback is given.

### Procedure

Via social media, participants were recruited for the event Smart Distance Lab: The Art Fair. Visitors were asked to purchase their ticket in advance. Upon purchasing a ticket, visitors were asked to participate in our study by filling out an informed consent form and a questionnaire on demographics and attitudes towards COVID-19. At the end of the questionnaire a code was provided that allowed participants to select a 30-min time slot to enter the event. In case people showed up at the art fair without having bought their ticket in advance, they could purchase a ticket directly without completing the questionnaire, and they were only asked to provide informed consent. If someone declined to participate in our study, they immediately received this code, and participation in our study was thus not required to visit the art fair. There was a separate code that allowed people to receive a free ticket, but the rest of the procedure was the same.

Visitors were allowed to enter the art fair during their selected time slot. Upon entering, it was explained that research was being carried out in the hall and a health check was conducted. At the Social Distance Sensor (SDS) handout station, visitors were asked whether they had signed the informed consent and completed the corresponding questionnaire. Visitors for whom this was not the case were asked whether they wanted to participate in the study and, if so, to sign the informed consent form. When visitors belonged to the same household, this was registered and the sensors were activated at the same time so that they would not report any incidents with each other. In the face mask condition, after handing out the sensors, face masks were handed out with the request to wear them until they were told that the masks could be removed. Visitors could then enter the art fair and stay inside for as long as they wanted, possibly taking part in multiple experimental conditions. On the way out, visitors handed in their sensors. In addition, they were asked to fill out a post questionnaire about their experience at the art fair.

The study was approved by the ethics review board of the University of Amsterdam (2020-CP-12488), and all participants provided informed consent before participating. All methods were performed in accordance with the relevant guidelines and regulations.

### Practical and technological problems

First, the face mask intervention was only executed on day 1, which allows us to only compare the face mask condition to the no supplementary intervention condition on day 1. Second, on day 3, two conditions were cut short (60 min instead of 120 min), see Table 1, and were excluded from analyses. Third, the buzzer notified visitors when they were within 1.5 m from another visitor, but the settings differed across days. Specifically, on the first day the buzzer only notified people after 2 s, which led to a lot of confusion. In addition, we did not actively demonstrate the buzzing feature to the visitors, which resulted in people trying out the feature inside the art fair. On day two and three, visitors were able to test out the buzzer before entering the art fair. In addition, on day two and three the buzzer notified the visitor immediately when they were within 1.5 m of another visitor, without the 2 s delay.

### Statistical analyses

#### Pre-processing and quality control of sensor data

The SDS output contained the reporting and opposing ID for each contact, the number of times they were within 1.5 m, and the time this contact was registered by the access point. Note that a single contact between two visitors is in this database twice, each visitor once as the ‘reporting ID’ and once as the ‘opposing ID’. First, we separated the output into the different experimental conditions and kept records that were between the start and end of each experimental condition (see Table 1 for start and finish times). Second, for each reporting ID in a condition, we checked whether the sensor was handed out during that condition as registered at the handout station. In case we could not link a sensor to a visitor, we removed this contact from the output. These records could occur when an SDS was activated but not handed out, e.g., when the SDS automatically activated when removed from the charger. Third, as the SDS output only contains records of contacts and the reporting and opposing IDs, we had to add visitors to the output who made zero contacts. More details on the processing of the data can be found in the Data Descriptor paper by Tanis et al.^28^.

#### Models

The contact networks of visitors were analyzed with a Bayesian logistic regression model including cross-nested random effects for the number of contacts of participants (or actors). For relation *Y*_*kij*_, between actors *i* and *j* in network *k*, the fitted model is:

$${\text{P}}\left( {Y_{kij} = 1} \right) = {{\exp \left( {\mu_{kij} } \right)} \mathord{\left/ {\vphantom {{\exp \left( {\mu_{kij} } \right)} {\left( {1 + \exp \left( {\mu_{kij} } \right)} \right)}}} \right. \kern-\nulldelimiterspace} {\left( {1 + \exp \left( {\mu_{kij} } \right)} \right)}}.$$

The log-odds $\mu_{kij}$ are constituted of an average *m*_*kij*_ and actor-specific random effects *A*_*ki*_ and *B*_*kj*_ which are assumed to a have normal distribution with a zero mean:

$$\mu_{kij} = m_{kij} + A_{ki} + B_{kj} ,\quad i \ne j.$$

The average *m*_*kij*_, consists of an overall mean *m*, and actor-level predictors *Xa*_*kij*_ with coefficient $\beta_{a}$:

$$m_{kij} = m + Xa_{kij} \beta_{a} .$$

This is a reduced version of the multilevel p_2_ model^32^ for non-directed networks, omitting reciprocity parameters, dyadic predictors, and random effects at the network level, and with identical random sender and receiver effects. In the current application, actor-level dummy variables were applied to model differences in the number of contacts between the two networks observed in different conditions. To distinguish this model from others, it was called b_2_ (b for bidirectional). Estimation was performed with Markov Chain Monte Carlo simulations, similar to the estimation of the j_2_ model^33,34^, with similar prior distributions. Code for this model is available in the R-package *dyads*^35^. We report posterior means as point estimates for the odds ratios in the main text together with their associated 95% credible interval. The credible interval gives the range within which the true odds ratio falls with 95% certainty.

## References

[1] WHO. Coronavirus disease (COVID-19) pandemic. https://www.who.int/emergencies/diseases/novel-coronavirus-2019. Retrieved December 31st.

[2] Van Bavel JJ (2020). Using social and behavioral science to support COVID-19 pandemic response. Nat. Hum. Behav..

[3] Chu DK (2020). Physical distancing, face masks, and eye protection to prevent person-to-person transmission of SARS-CoV-2 and COVID-19: A systematic review and meta-analysis. Lancet.

[4] Haug N (2020). Ranking the effectiveness of worldwide COVID-19 government interventions. Nat. Hum. Behav..

[5] Flaxman S (2020). Estimating the effects of non-pharmaceutical interventions on COVID-19 in Europe. Nature.

[6] Howard J (2021). An evidence review of face masks against COVID-19. Proc. Natl. Acad. Sci. U.S.A..

[7] Lazzarino AI, Steptoe A, Hamer M, Michie S (2020). COVID-19: Important potential side effects of wearing face masks that we should bear in mind. BMJ.

[8] World Health Organization. *Advice on the Use of Masks in the Context of Covid-19: Interim Guidance*. 1 Dec 2020. https://www.who.int/publications-detail/advice-on-the-use-of-masks-in-the-community-during-home-care-and-in-healthcare-settings-in-the-context-of-the-novel-coronavirus-(2019-ncov)-outbreak.

[9] Prasad V, Jena AB (2014). The Peltzman effect and compensatory markers in medicine. Healthcare.

[10] Betsch C (2020). Social and behavioral consequences of mask policies during the COVID-19 pandemic. Proc. Natl. Acad. Sci. U.S.A..

[11] Martin GP, Hanna E, McCartney M, Dingwall R (2020). Science, society, and policy in the face of uncertainty: Reflections on the debate around face coverings for the public during COVID-19. Crit. Public Health.

[12] Marchiori, M. *COVID-19 and the Social Distancing Paradox: Dangers and solutions*. Preprint at https://arxiv.org/abs/2005.12446 (2020).

[13] Seres, G. *et al*. *Face Masks Increase Compliance with Physical Distancing Recommendations During the COVID-19 Pandemic*. Preprint at https://osf.io/es7kt/ (2020).

[14] Seres, G., *et al*. Face mask use and physical distancing before and after mandatory masking: Evidence from public waiting lines. Preprint at 10.2139/ssrn.3641367 (2020). PMC8604556 34840368

[15] Aranguren M (2021). Face mask use conditionally decreases compliance with physical distancing rules against COVID-19: Gender differences in risk compensation pattern. Ann. Behav. Med..

[16] IJzerman H (2020). Use caution when applying behavioral science to policy. Nat. Hum. Behav..

[17] Heffner J, Vives ML, FeldmanHall O (2020). Emotional responses to prosocial messages increase willingness to self-isolate during the COVID-19 pandemic. Pers. Individ. Differ..

[18] Pfattheicher S (2020). The emotional path to action: Empathy promotes physical distancing during the COVID-19 pandemic. Psychol. Sci..

[19] Capraro V, Barcelo H (2021). Telling people to “rely on their reasoning” increases intentions to wear a face covering to slow down COVID-19 transmission. Appl. Cognit. Psychol..

[20] Banker S, Park J (2020). Evaluating prosocial COVID-19 messaging frames: Evidence from a field study on Facebook. Judgm. Decis. Mak..

[21] Bilancini E (2020). The effect of norm-based messages on reading and understanding COVID-19 pandemic response governmental rules. JBEP.

[22] Capraro V, Barcelo H (2020). The effect of messaging and gender on intentions to wear a face covering to slow down COVID-19 transmission. JBEP.

[23] Lunn PD (2020). Motivating social distancing during the Covid-19 pandemic: An online experiment. Soc. Sci. Med..

[24] Borsboom, D., *et al.* The lighting of the BECONs: A behavioral data science approach to tracking interventions in COVID-19 research. Preprint at 10.31234/osf.io/53ey9.

[25] Van Duijn, M. A. Estimation of a random effects model for directed graphs. In *SSS’95. Symposium Statistische Software* (ed. Snijders, T. A. B.) 113–131 (1995).

[26] Hoeben EM (2021). Social distancing compliance: A video observational analysis. PLoS ONE.

[27] Iyengar KP (2021). Influence of the Peltzman effect on the recurrent COVID-19 waves in Europe. Postgrad. Med. J..

[28] Tanis CC (2020). The Smart Distance Lab’s art fair, experimental data on social distancing during the COVID-19 pandemic. Sci. Data.

[29] Duives DC, Daamen W, Hoogendoorn SP (2013). State-of-the-art crowd motion simulation models. Transp. Res. Part C Emerg. Technol..

[30] Morwitz VG, Fitzsimons GJ (2004). The mere-measurement effect: Why does measuring intentions change actual behavior?. J. Consum. Psychol..

[31] Richter G, Raban DR, Rafaeli S, Reiners T, Wood LC (2015). Studying gamification: The effect of rewards and incentives on motivation. Gamification in Education and Business.

[32] Zijlstra BJ, Van Duijn MA, Snijders TA (2006). The multilevel p_2_ model A random effects model for the analysis of multiple social networks. Methodology.

[33] Zijlstra BJ (2017). Regression of directed graphs on independent effects for density and reciprocity. J. Math. Sociol..

[34] Van Duijn MA, Snijders TA, Zijlstra BJ (2004). p2: A random effects model with covariates for directed graphs. Stat. Neerl..

[35] Bonne J.H. Zijlstra (2020). dyads: Dyadic Network Analysis. R package version 1.1.3. https://CRAN.R-project.org/package=dyads.

